# Non-high-density lipoprotein cholesterol (non-HDL-C) levels in children with nonalcoholic fatty liver disease (NAFLD)

**DOI:** 10.1186/2193-1801-3-407

**Published:** 2014-08-05

**Authors:** Naim Alkhouri, Katharien Eng, Rocio Lopez, Valerio Nobili

**Affiliations:** Department of Pediatric Gastroenterology, Cleveland Clinic, 9500 Euclid Avenue, A111, Cleveland, OH 44195 USA; Digestive Disease Institute, Cleveland Clinic, 9500 Euclid Avenue, A51, Cleveland, OH 44195 USA; Quantitative Health Sciences, Cleveland Clinic, 9500 Euclid Avenue, Cleveland, OH 44195 USA; Liver Unit, Bambino Gesù Children’s Hospital and Research Institute, 37 Salita di Sant’onofrio, 00165 Rome, Italy

**Keywords:** Non-alcoholic fatty liver disease, Non-alcoholic steatohepatitis, Children, Non-high-density lipoprotein cholesterol, Histology, Atherosclerosis, Cardiovascular disease risk

## Abstract

Non-alcoholic fatty liver disease (NAFLD) is associated with increased cardiovascular disease (CVD) risk in children. Non-high density lipoprotein-cholesterol (non-HDL-C) has been shown to be a good predictor of cardiovascular events. Recent data in adults found non-alcoholic steatohepatitis (NASH) to be associated with significantly higher levels of non–HDL-C than simple steatosis, suggestive it might be used as a non-invasive tool to diagnose NASH. The goal of our study was to assess non-HDL-C levels in children with NAFLD. Our cohort consisted of pediatric patients with biopsy-proven NAFLD. Anthropometric, laboratory, and histologic data were obtained on all patients. Univariable analysis was performed to assess differences in clinical characteristics between groups. Spearman rank correlation coefficients were calculated to assess the correlation between non-HDL-C levels and clinical variables. ANCOVA was used to adjust for possible confounders. 302 subjects with NAFLD were included in our study; 203 with NASH and 99 without NASH. Subjects with NASH had significantly higher non-HDL-C levels than those without (p = 0.004). Histologic features of NASH, including ballooning, inflammation, and fibrosis were found to be weakly correlated with non-HDL-C levels, (p < 0.05 for all). After adjusting for the presence of metabolic syndrome (MetS), ALT, and GGT, the association between non-HDL-C and NASH was not significant (p = 0.66). In Conclusion, non-HDL-C levels are higher in children with NASH than those with simple steatosis, suggesting increased CVD risk. This may be a reflection of the higher prevalence of MetS. Non-HDL-C had a positive association with histologic features of NASH.

## Introduction

The incidence of nonalcoholic fatty liver disease (NAFLD) has been increasing dramatically over the past three decades, which has been in parallel with the childhood obesity epidemic. NAFLD is now the most common cause of chronic liver disease in children and adolescents (Schwimmer et al. 2006; Wieckowska and Feldstein 2005; Chan et al. 2004). NAFLD encompasses a spectrum of diseases that ranges from simple steatosis (characterized by hepatic lipid accumulation) to non-alcoholic steatohepatitis (NASH) (characterized by evidence of lipid accumulation with associated hepatocyte inflammation, injury, and varying degrees of fibrosis), which can progress to cirrhosis and even end-stage liver disease (Vajro et al. 2012; Feldstein et al. 2009). Multiple studies are suggesting that NAFLD may be the hepatic manifestation of metabolic syndrome (MetS) (Abdelmalek and Diehl 2007; Pagano et al. 2002). There appears to be a strong association between NAFLD, MetS, and insulin resistance, which has brought into question the possibility of NAFLD playing a role in cardiovascular disease (CVD). Furthermore, recent evidence has now linked NAFLD with an increased prevalence of CVD, independent of the presence of MetS or traditional risk factors (Alkhouri et al. 2011; Targher 2007; Targher et al. 2008a). More importantly, it has also been found that children with NASH can be at an even higher risk for atherosclerosis than those patients with simple steatosis (Nobili et al. 2010).

Recently, non-high-density lipoprotein cholesterol (non-HDL-C) has become increasingly recognized as an important measure of atherogenic particles, as it reflects the cholesterol in all lipoprotein particles that are considered to be atherogenic. Non-HDL-C has been strongly associated with predicting coronary artery disease (Orakzai et al. 2009). In adults, it has been added on to a recommended screening algorithm by The Adult Treatment Panel III of the National Cholesterol Education Program (Grundy et al. 2004). Additionally, in a consensus report by the American Diabetes Association and American College of Cardiology Foundation, they identified that non-HDL-C was a better marker than low-density lipoprotein (LDL) cholesterol for identifying high-risk patients at cardiovascular risk (Brunzell et al. 2008).

Taking into consideration that patients with NAFLD are at increased cardiovascular risk, and that those patients with NASH may be at even higher risk for atherosclerosis than those with simple steatosis, a recent adult study investigated the ability of non-HDL-C to differentiate NASH from simple steatosis in patients with NAFLD. In those patients who did not take any lipid-lowering agents, NASH was associated with significantly higher levels of non-HDL-C as compared to those patients with just simple steatosis. Their study concluded that non-HDL-C could be utilized as a non-invasive biomarker to differentiate between steatosis and NASH (Corey et al. 2012). In our study we aimed to examine non-HDL-C levels in pediatric patients with NAFLD to assess if increased CVD risk may be associated with worsening histologic severity of NAFLD, and more specifically NASH as compared to simple steatosis.

## Materials and methods

### Patients

Pediatric patients with biopsy-proven NAFLD were seen at and enrolled in this study performed at the Bambino Gesù Children’s Hospital (Rome, Italy) between January 2003 and December 2009. This study was approved by the ethics committee of the Bambino Gesù Children’s Hospital and Research Institute. The study was carefully explained to the children’s guardians and written consent was obtained.

All included subjects raised suspicion for NAFLD based on persistently elevated serum aminotransferase levels and imaging studies that revealed a diffusely hyperechogenic liver suggestive of fatty liver, with a final diagnosis of NAFLD made on liver biopsy. Exclusion criteria for these patients with NAFLD including (1) alcohol consumption; (2) hepatic viral infections (such as hepatitis A, B, C, D, and E; cytomegalovirus; and Epstein-Barr virus); (3) history of parenteral nutrition; (4) use of drugs that are known to induce steatosis (e.g.: prednisone, valproate, or amiodarone), affect body weight or carbohydrate metabolism; and (5) known liver disease, such as autoimmune liver disease, metabolic liver disease, alpha-1-antitrypsin associated liver disease, and Wilson’s disease. These were ruled out using standard clinical, laboratory, and histological criteria.

### Anthropometric data

Clinical variables were recorded, which included standard procedures for height and weight (Lohman et al. 1988). The body mass index (BMI) and its standard deviation score (Z score) were calculated (Kuczmarski et al. 2000). Obesity was defined by a BMI greater than or equal to 95^th^ percentile adjusted for age and sex. Waist circumference was also measured, which was evaluated at the highest point of the iliac crest with a standing subject (Li et al. 2006). Waist circumference to height ratio was also calculated.

MetS in this cohort was defined as having three or more of the following five criteria: (1) abdominal obesity, defined as waist circumference (WC) ≥ 90^th^ percentile for age (Fernandez et al. 2004); (2) low HDL-cholesterol, defined as concentrations <5^th^ percentile for age and sex (American Academy of Pediatrics 1992); (3) hypertriglyceridemia, defined as triglyceride (TG) level >95^th^ percentile for age and sex (American Academy of Pediatrics 1992); (4) hypertension, defined as systolic or diastolic blood pressure >95^th^ percentile for age and sex (Report of the Second Task Force on Blood Pressure Control in Children 1987); and (5) impaired fasting glucose (≥110 mg/dL), impaired glucose tolerance or known type 2 diabetes mellitus (Genuth et al. 2003).

### Laboratory assessment

Laboratory data collection included total cholesterol (TC), HDL cholesterol, triglycerides, aspartate aminotransferase (AST), alanine aminotransferase (ALT), serum γ-glutamyltransferase (GGT), total bilirubin, albumin, and INR. These were obtained and measured by standard laboratory methods. The non-HDL-C was calculated from the total cholesterol subtracted by the HDL cholesterol (non-HDL-C = TC – HDL). The homeostasis model assessment of insulin resistance (HOMA-IR) (Matthews et al. 1985) was calculated as markers for insulin sensitivity. None of the patients were on lipid-lowering medications at the time of the lipid profile measurements.

### Liver histology

Liver biopsy was performed in all subjects because of the clinical indication to either assess the presence of NASH, degree of fibrosis, and/or to identify if other liver diseases were present. After an overnight fast, liver biopsy was performed under general anesthesia and ultrasound guidance utilizing the automatic core biopsy 18 gauge needle (Biopince, Amedic, Sweden). The Sonoline Omnia ultrasound machine (Siemens, Munich, Germany) was used, which is equipped with a 5-MHz probe (5.0 C 50, Siemens) and biopsy adaptor. For each subject, two biopsy passes in different liver segments were made, and the length of the liver specimen was recorded in millimeters. Only samples that included at least 5–6 complete portal tracts and with a length of ≥ 15 mm (Poynard et al. 2007) met the requirements for this study. Biopsies were routinely processed and staining of liver tissue included hematoxylin-eosin, Periodic acid-Schiff diastase, Van Gieson, and Prussian blue stain. A single hepatopathologist reviewed the biopsies and was blinded to the clinical and laboratory data.

Biopsies were evaluated by a single expert pediatric hepatopathologist who established the histopathological diagnosis of NASH. Based on this categorization patients were divided into two groups: 1) “NASH” or 2) diagnosis not compatible with NASH or “not NASH”. Liver histology was scored using the NAFLD activity scoring (NAS) system developed by the NASH Clinical Research Network (Kleiner et al. 2005). The grade of steatosis (0 – 3), hepatocyte ballooning (0 – 2), and lobular inflammation (0 – 3) were totaled to determine the NAS (0 – 8) score. Portal inflammation (PI) was graded from 0 to 2 (0 = no PI, 1 = mild PI, and 2 = more than mild PI). Mild PI was defined as a few mononuclear cells, usually in more than one portal tract. More than mild PI was defined as at least one portal area showing moderate to marked density of inflammation, and/or the presence of lymphoid aggregates. Fibrosis was staged as the following: 0 = no fibrosis, 1 = periportal or perisinusoidal, 2 = perisinusoidal and portal/periportal fibrosis, 3 = bridging fibrosis, and 4 = cirrhosis.

### Statistical analysis

Descriptive statistics were performed for all variables. Categorical variables were presented as frequencies and percentages. Continuous variables were presented as mean ± standard deviation or median [25^th^, 75 percentiles]. Univariable analysis was performed to assess differences between subjects with and without NASH. Student’s t-tests or the non-parametric Wilcoxan rank sum tests were used to compare continuous or ordinal variables. Pearson’s chi-square tests were used for categorical factors. Spearman rank correlation coefficients (rho) were calculated to assess the correlation between non-HDL cholesterol levels and liver histology features (steatosis, ballooning, inflammation, and degree of fibrosis) and clinical characteristics. Analysis of covariance (ANCOVA) was used to adjust for possible confounders. All demographic and clinical factors were considered for inclusion in the model, and an automated stepwise variable selection was performed on 1,000 bootstrap samples to choose the final model. Variables with inclusion rates of at least 50% were included in the model. A p < 0.05 was considered statistically significant. All analyses were performed using SAS (version 9.2, The SAS Institute, Cary, NC) and R (version 2.13.1, The R Foundation for Statistical Computing, Vienna, Austria).

## Results

### Demographic and clinical characteristics

Three hundred and two children with biopsy-proven NAFLD were included in this study. Table 1 summarizes the anthropometric, clinical and laboratory data. There were 203 subjects with biopsy proven NASH (67.2%) and 99 subjects without NASH (32.8%). Of the total subjects, 36.4% were male, 63.6% were female. The mean age at initial visit for this study was 12.3 ± 3.1 years. Two hundred and sixty-eight (88.7%) children were found to be obese. One hundred and sixty-eight (55.6%) children were found to have MetS. Univariable analysis was performed, which showed that obesity, greater WC, hypercholesterolemia, hypertriglyceridemia, MetS, and higher non-HDL cholesterol were significantly associated with NASH. Total cholesterol and triglyceride levels were significantly higher in NASH patients; whereas, HDL-C levels were similar in the two groups. In addition, AST, GGT, and lower total bilirubin were also significantly associated with NASH.

**Table 1:** **Demographic and clinical characteristics of subjects**

Factor	NASH (N = 203)	Not NASH (N = 99)	p-value
**Sex**			
**Male**	68 (33.5)	42 (42.4)	0.13
**Age at first visit** ***(y)***	12.3 ± 3.1	12.3 ± 3.1	0.98
**Obese**	188 (92.6)	80 (80.8)	***0.002***
**BMI** ***(kg/m*** ^***2***^ ***)***	26.3 ± 4.2	25.5 ± 3.7	0.11
**BMI percentile**	97.0 [94.0, 98.0]	96.0 [92.0, 98.0]	0.12
**Waist circumference** ***(cm)***	91.7 ± 11.4	89.1 ± 9.5	***0.044***
**WC percentile**	97.0 [90.0, 97.0]	90.0 [89.0, 97.0]	***<0.001***
**WC/Height ratio**	0.60 ± 0.04	0.58 ± 0.04	***<0.001***
**Total cholesterol** ***(mg/dL)***	163.5 ± 32.6	149.2 ± 25.3	***<0.001***
**HDL** ***(mg/dL)***	53.0 [39.0, 69.0]	56.0 [38.0, 71.0]	0.96
**Non-HDL cholesterol** ***(mg/dL)***	105.8 ± 40.2	92.3 ± 32.8	***0.004***
**Triglycerides** ***(mg/dL)***	114.0 ± 62.9	84.6 ± 41.8	***<0.001***
**Hypercholesterolemia**	121 (59.6)	41 (41.4)	***0.003***
**Hypertriglyceridemia**	136 (67.0)	52 (52.5)	***0.015***
**Hypertension**	55 (27.1)	29 (29.3)	0.69
**HOMA-IR**	2.7 ± 1.7	2.6 ± 2.0	0.83
**IGT/Diabetes**	88 (43.3)	45 (45.5)	0.73
**Metabolic syndrome**	131 (64.5)	37 (37.4)	***<0.001***
**ALT** ***(U/L)***	73.0 [50.0, 99.0]	70.0 [50.0, 89.0]	0.41
**AST** ***(U/L)***	57.5 ± 29.3	49.9 ± 16.3	***0.017***
**GGT** ***(U/L)***	27.0 [20.0, 40.0]	25.0 [17.0, 33.0]	***0.031***
**Total bilirubin** ***(mg/dL)***	0.65 ± 0.24	0.73 ± 0.22	***0.007***
**Albumin** ***(g/dL)***	4.6 ± 0.53	4.6 ± 0.73	0.64

Values presented as Mean ± SD with t-test; Median [P25, P75] with Wilcoxon rank sum test, or N (%) with Pearson’s chi-square test.

P-values in bold are statistically significant.

### Liver biopsy findings

Histologic features for subjects are represented in Table 2. As expected, subjects with NASH had higher NAS scores than those without. Majority of subjects with NASH had mild (134 subjects; 66%) or more than mild (21 subjects; 10.3%) portal inflammation, and ballooning was present in 135 subjects (66.5%). Lastly, in those patients with NASH, some degree of fibrosis was noted in majority of patients: mild fibrosis (grade 1) in 137 patients (67.5%); moderate fibrosis (grade 2) in 15 patients (7.4%); and advanced fibrosis (grade 3) in 18 patients (8.9%). None of these patients had liver cirrhosis. The mean NAS score in patients with NASH was 4.5 ± 1.4. In those patients without NASH, NAS was 2.2 ± 0.65, p <0.001.

**Table 2:** **Histologic features**

Factor	NASH (N = 203)	Not NASH (N = 99)	p-value
**Steatosis**			***<0.001***
**<5%**	0 (0.0)	2 (2.0)	
**5-33%**	34 (16.7)	68 (68.7)	
**34-65%**	85 (41.9)	26 (26.3)	
**> = 66%**	84 (41.4)	3 (3.0)	
**Lobular inflammation**			***<0.001***
**None**	5 (2.5)	19 (19.2)	
**<2 under 20×**	147 (72.4)	80 (80.8)	
**2–4 under 20×**	48 (23.6)	0 (0.0)	
**>4 under 20×**	3 (1.5)	0 (0.0)	
**Portal inflammation**			***<0.001***
**None**	48 (23.6)	55 (55.6)	
**Mild**	134 (66.0)	43 (43.4)	
**More than mild**	21 (10.3)	1 (1.0)	
Ballooning*			***<0.001***
**None**	66 (32.8)	94 (94.9)	
**Few**	55 (27.4)	5 (5.1)	
**Many**	80 (39.8)	0 (0.0)	
**Fibrosis**			***<0.001***
**0**	33 (16.3)	75 (75.8)	
**1**	137 (67.5)	18 (18.2)	
**2**	15 (7.4)	4 (4.0)	
**3**	18 (8.9)	2 (2.0)	
NAS*	4.5 ± 1.4	2.2 ± 0.65	***<0.001***

*Data not available for all subjects. Missing values: Ballooning = 2, NAS = 2.

Values presented as Mean ± SD with t-test or N (%) with Wilcoxon rank sum tests.

P-values in bold are statistically significant.

### Non-HDL-C levels were higher in the NASH cohort and had positive correlation with histological features

**Figure 1:**
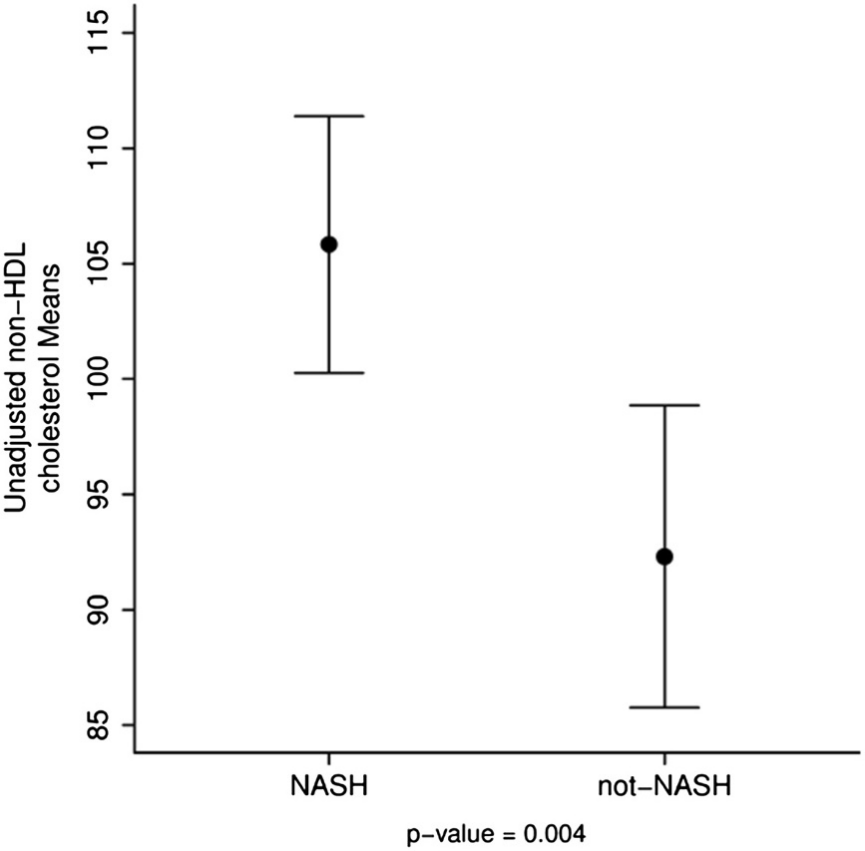
**Unadjusted non-HDL cholesterol levels.**

**Figure 2:**
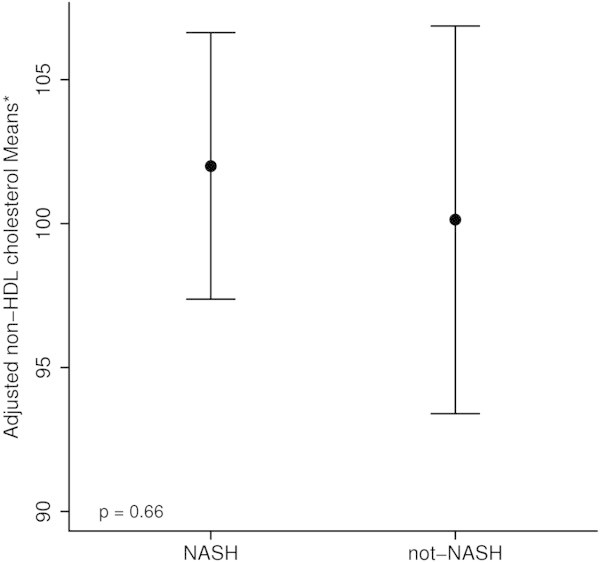
**Adjusted non-HDL cholesterol levels.**

Subjects with NASH were found to have significantly higher non-HDL-C levels than those without NASH (105.8 ± 40.2 versus 92.3 ± 32.8; p = 0.004) (Figure 1). Importantly, histologic features of pediatric NASH including lobular inflammation, portal inflammation, and ballooning had a positive correlation with non-HDL-C levels that was statistically significant (p = 0.005, p = 0.001, p = 0.01 respectively), and the NAS score was significantly associated with non-HDL-C (p = 0.024) as shown in Table 3. Moreover, fibrosis was also found to be positively correlated to non-HDL-C levels (Spearman’s rho of 0.25, 95% CI: 0.14, 0.36; p <0.001). The grade of steatosis, on the other hand, was not significantly associated with non-HDL-C. The association between non-HDL-C level and the histologic diagnosis of NASH was not significant after adjusting for MetS, WC/Height ratio, GGT and ALT in the multivariable analysis (Figure 2) (adjusted mean (95% CI): 102 (97.4, 106.6) vs 100.1 (93.4, 106.9); p = 0.66) implicating that other factors known to be associated with increased cardiovascular risk may explain the association noted in the univariable analysis.

**Table 3:** **Correlations between non-HDL cholesterol levels and histologic features**

Factor	Spearman’s rho	95% CI	p-value
**Steatosis**	0.02	(−0.09, 0.13)	0.71
**Lobular inflammation**	0.16	(0.05, 0.27)	0.005
**Portal inflammation**	0.19	(0.07, 0.30)	0.001
**Ballooning**	0.15	(0.04, 0.26)	0.01
**Fibrosis**	0.25	(0.14, 0.36)	<0.001
**NAS**	0.13	(0.02, 0.24)	0.024

rho: correlation coefficient; CI: confidence interval.

## Discussion

In this large study of pediatric patients with biopsy-proven NAFLD, our main findings have demonstrated that (1) non-HDL-C levels were higher in children with biopsy proven NASH as compared to children with simple steatosis and (2) histologic features of NASH, such as ballooning, lobular inflammation, portal inflammation and fibrosis were found to be correlated with non-HDL-C levels. This data has further extended the knowledge base of the cardiovascular complications of NASH in the pediatric population with NAFLD. The higher-non-HDL-C levels found in this study adds to prior studies that have shown an increased risk of CVD in patients with NASH. Interestingly, on multivariable analysis this finding did not hold when adjusting for MetS, WC/Height ratio (a measure of central obesity and associated with MetS) (Bener et al. 2013), GGT, and ALT. Given the lack of a significant difference of non-HDL-C in those patients with and without NASH on multivariable analysis, our study does not support non-HDL-C as a single biomarker for NASH. The etiology of this finding on multivariable analysis remains unclear, and while it may be more of a reflection of the increased prevalence of MetS in NASH, there is also a likely component of increased hepatic inflammation given the role that GGT and ALT have in this study’s multivariable analysis.

Studies in both adults and children have shown an association between NAFLD and CVD (Alkhouri et al. 2011; Targher 2007; Schwimmer et al. 2008). In one case control study of 150 overweight children with biopsy proven NAFLD versus those without, NAFLD was found to be strongly associated with risk factors for cardiovascular disease, including higher glucose, insulin, blood pressure, TC, LDL, triglyceride, and lower HDL. Furthermore, obese children with NAFLD were significantly more likely to have metabolic syndrome as compared to obese children without NAFLD (Schwimmer et al. 2008). Various adult studies have also looked at diagnostic markers for early atherosclerosis, which has included findings of a significant increase in carotid intima-media thickness (CIMT) in patients with NAFLD (Brea et al. 2005), with some CIMT studies even suggesting NAFLD to be an independent risk factor for developing atherosclerosis (Mohammadi et al. 2011; Li et al. 2012). Interestingly, while one pediatric study did not show CIMT to be associated with NAFLD in children (Manco et al. 2010), a recent pediatric study in 2012 showed CIMT to be significantly higher in patients with NAFLD as compared to obese and normal controls (Gokce et al. 2013). Other studies have also found impaired endothelial function and increased arterial stiffness in patients with NAFLD (Vlachopoulos et al. 2010; Villanova et al. 2005).

There continues to be a growing body of evidence that has shown an increased prevalence of CVD risk in patients with NAFLD, with additional studies showing that it can be independent of the presence of MetS or traditional risk factors (Alkhouri et al. 2011; Targher 2007; Targher et al. 2008a). A recent adult study found lipid ratios to be significantly associated with NAFLD, with a stepwise statistically significant difference between patients who had normal biopsies versus simple steatosis versus NASH. But more interestingly, the histologic severity of inflammation and liver injury appeared to be strongly associated with a more atherogenic lipid profile and increased CVD (Alkhouri et al. 2010). The atherogenic risk of NAFLD can be found not only in the adult population, but also in the pediatric population, with increased risk with progression of disease. A prior pediatric study from our institution evaluated 118 consecutive children with biopsy-proven NAFLD and found that the severity of liver injury and fibrosis was strongly associated with certain markers of atherogenic risk, in particular, ratios of cholesterol ester-rich lipoproteins such as TC/HDL, LDL/HDL, and TG/HDL. These lipid ratios were found to have a significant positive correlation with the NAFLD activity and fibrosis scores, with also a significant difference found between those with definitive NASH as compared to those with simple steatosis or borderline diagnosis. Additionally, this association was found to be independent of the presence of MetS, insulin resistance, and obesity (Nobili et al. 2010). In our current study we investigated CVD risk in obese children through non-HDL-C levels, which were found to be significantly higher in children with biopsy proven NASH as compared to those with simple steatosis. In addition, histologic features of NASH were also found to be correlated with non-HDL-C levels.

Potential biological mechanisms for a higher risk for atherosclerosis in NAFLD have been described, which includes increased systemic inflammation and oxidative stress. Markers, such as C-reactive protein (CRP) and fibrinogen levels, which are known to be inflammatory markers, are increased in patients with NAFLD, and particularly in those with NASH (Yoneda et al. 2007; Targher et al. 2008b). In our study, the significant difference of non-HDL-C in those patients with NASH versus simple steatosis highlights the cardiovascular implications of NASH. But in addition, the positive correlation of non-HDL-C levels with histologic scores of ballooning, inflammation, and fibrosis in NASH speaks towards the potential relationship between severity of hepatic damage and an atherogenic lipid profile.

Hepatic inflammation in it of itself is thought to be atherogenic, as studies have found a strong association between CVD and elevated ALT and GGT, which are surrogate markers of inflammation (Schindhelm et al. 2007; Lee et al. 2007; Ioannou et al. 2006). Recent animal studies have also suggested that hepatic insulin resistance and hepatic inflammation can also contribute to the development of dyslipidemia and increased cardiovascular disease risk (Guillen et al. 2008; Biddinger et al. 2008). In our study, the significance of non-HDL-C as a distinguishing marker for NASH on multivariate analysis was lost when controlling for such serum markers as GGT and ALT, which themselves are markers of inflammation and associated with cardiovascular risk.

It is also hypothesized that alterations in lipid metabolism may influence the development of steatosis versus NASH. Some recent studies have discussed the role of very low density lipoprotein (VLDL) and triglyceride production in the development of MetS and NASH (Choi and Ginsberg 2011; Choi and Diehl 2008; Fujita et al. 2009; Adiels et al. 2008). It is additionally thought that alterations in the exportation of triglycerides (packaged as VLDL and intermediate density lipoprotein (IDL)) may influence hepatic lipid storage and the development of NAFLD (Fujita et al. 2009; Choi et al. 2007; Yamaguchi et al. 2007). Unfortunately, direct measures of VLDL and IDL are not readily available to clinicians and are expensive. However, non-HDL-C is an indirect measure that encompasses both VLDL and IDL along with other lipoproteins, which include LDL, lipoprotein A, and chylomicrons. Our findings in this study may indirectly highlight the potential influence that VLDL and IDL may have on development of steatosis versus NASH.

The main strength of this study is the inclusion of a large group of over 300 children with biopsy-proven NAFLD that includes a full spectrum of the disease and varying degrees of fibrosis. None of the children in the current study were on lipid lower agents at the time of lipid measurement. In addition, there was an extensive characterization of anthropometric, metabolic, and histologic profile of these patients. To our knowledge, this is the first pediatric study to look at non-HDL-C in patients with NAFLD. However, this study has some limitations, which includes the fact that our patients were seen at a large referral tertiary care medical center, with a majority of our NAFLD patients having biopsy-proven NASH and a high prevalence rate of fibrosis. These findings therefore may not be generalizable to the pediatric population in the community. In addition, the majority of children were of Caucasian descent, thus the association between NAFLD and non-HDL-C may be different among other ethnicities. Lastly, this study is a cross-sectional study, thus it only showed an association between the severity of NAFLD and non-HDL-C, and thus could not infer causation.

In conclusion, non-HDL-C levels were found to be higher in children with NASH as compared to those with simple steatosis, suggestive of an increased risk for CVD. This may be more of a reflection of an increased prevalence of MetS in this population, but the increased hepatic inflammation of NASH may have played a role in the finding. Histologic features of NASH, such as ballooning, inflammation, and fibrosis were found to be correlated with non-HDL-C levels. Together, this continues to highlight again the association of NASH with increased CVD risk. Future studies would be beneficial to further evaluate the role of non-HDL-C and its association with increased CVD risk in children with NAFLD.

### Informed consent and ethics committee approval

Informed consent was obtain in all patients. This study was approved by the responsible ethics committee of the Bambino Gesù Children’s Hospital and Research Institute.

### Compliance with ethical requirements

All authors declare no conflict of interest for this study.

All procedures were followed in accordance with the ethical standards of the responsible committee on human experimentation (institutional and national) and with the Helsinki Declaration of 1975, as revised in 2008.

Informed consent was obtained from all patients included in this study.

## Abbreviations

NAFLD: Nonalcoholic fatty liver disease
NASH: Non-alcoholic steatohepatitis
non-HDL-C: Non-high density lipoprotein cholesterol
NAS: NAFLD activity score
CVD: Cardiovascular disease
MetS: Metabolic syndrome
BMI: Body mass index
WC: Waist circumference
HOMA-IR: Homeostasis model assessment index of insulin resistance
IGT: Impaired glucose tolerance
TG: Triglycerides
IDL: Intermediate density lipoprotein
LDL: Low density lipoprotein
AST: Aspartate aminotransferase
ALT: Alanine aminotransferase
GGT: Serum γ-glutamyltransferase
INR: International normalized ratio
ANCOVA: Analysis of covariance
CIMT: Carotid intima-media thickness
PI: Portal inflammation.
